# Patterns of the Circulation of Influenza in a Targeted Jordanian Subpopulation from November 2021 to April 2023

**DOI:** 10.3390/pathogens14040365

**Published:** 2025-04-08

**Authors:** Ashraf I. Khasawneh, Nisreen M. Himsawi, Jumana A. Abu-Raideh, Ashraf Sammour, Hazem Abu Safieh, Mohammad Al Qudah, Ali Obeidat, Moureq R. Alotaibi, Hafez Al-Momani, Rame Khasawneh, Sofian Al Shboul, Tareq Saleh

**Affiliations:** 1 Department of Microbiology, Pathology, and Forensic Medicine, Faculty of Medicine, The Hashemite University, Zarqa 13133, Jordan; 2 Department of Anatomy, Physiology & Biochemistry, Faculty of Medicine, The Hashemite University, Zarqa 13133, Jordan; 3 Department of Pathology and Microbiology, Faculty of Medicine, Jordan University of Science and Technology, Irbid 21110, Jordan; 4 Department of Otorhinolaryngology, Irbid Specialty Hospital, Irbid 21110, Jordan; 5 Department of Pharmacology and Toxicology, College of Pharmacy, King Saud University, Riyadh 11481, Saudi Arabia; 6 King Hussein Medical Center, Royal Medical Services, Amman 11942, Jordan; 7 Department of Pharmacology and Public Health, Faculty of Medicine, The Hashemite University, Zarqa 13133, Jordan; 8 Department of Pharmacology & Therapeutics, College of Medicine & Health Sciences, Arabian Gulf University, Manama P.O. Box 26671, Bahrain

**Keywords:** influenza A, influenza B, epidemiology, coinfections, (H1N1) pdm09, Victoria lineage, Jordan

## Abstract

Background: Influenza remains a global health challenge, causing significant morbidity and mortality. This study explores the epidemiology of influenza A (IAV) and B (IBV) during the 2021–2023 winter seasons within a targeted Jordanian subpopulation to inform public health strategies. Methods: Nasopharyngeal swabs from patients with acute respiratory tract infections (ARTIs) in three major Jordanian cities were analyzed. RT-PCR was utilized to detect common respiratory pathogens, and specific primers identified IAV (H1N1) pdm09, H3N2, and IBV subtypes. Statistical analyses examined influenza subtype frequencies and their association with demographics and coinfection patterns. Results: IAV, IBV, and ICV were detected in 9.4%, 13.5%, and 5.5% of cases, respectively. Predominant strains were IAV (H1N1) pdm09 (55.8%), H3N2 (30.2%), and IBV Victoria lineage (98.4%). Coinfections with IAV frequently involved *Bordetella* spp., *Staphylococcus aureus*, and IBV, while IBV also showed coinfections with *Haemophilus influenzae* type B and IAV. Conclusions: The predominance of IAV (H1N1) pdm09 and IBV Victoria lineage highlights the need for strain-specific vaccination. Frequent coinfections underscore the importance of comprehensive diagnostics. Local public health strategies should focus on increasing vaccine coverage and preventive education, especially for adults and urban populations.

## 1. Introduction

Influenza remains a significant public health concern in Jordan, particularly in densely populated urban areas. The country’s geographical location and climate contribute to seasonal variations in influenza transmission, with outbreaks typically occurring during the colder months (from December to March). Influenza A(H1N1) pdm09, which emerged as part of the 2019 pandemic, has since become the most prevalent influenza A subtype in Jordan [1]. Similarly, the A(H3N2) subtype, was found to be a major driver of seasonal influenza activity [1]. Evidently, a study by Rolsma et al. (2021) [2] analyzing influenza cases from 2010 to 2013 found that influenza A accounted for the majority of infections, with A(H1N1) pdm09 accounting for 29.4% of the cases, A(H3N2) for 26.9%, influenza B for 23.5%, and influenza C for 16.0%.

Influenza B, though less common than influenza A, has also contributed to seasonal outbreaks in Jordan. The Victoria lineage has been more frequently detected, particularly among children and in years when influenza A activity is lower [2,3]. Unlike influenza A, which undergoes significant antigenic variation leading to annual shifts in circulating strains, influenza B exhibits more stable epidemiological patterns. Importantly, the co-circulation of influenza A and B presents challenges for surveillance and vaccine development, particularly given the suboptimal vaccination coverage in Jordan. Ongoing research continues to evaluate the effectiveness of vaccines against both influenza A and B [4,5].

The epidemiology of influenza in Jordan mirrors trends observed across the Middle East and North Africa (MENA), where influenza transmission is shaped by a combination of viral circulation, climate factors, and demographic patterns. Influenza A, particularly A(H1N1) pdm09, has remained the dominant circulating strain between 2009 and 2017, while influenza B, especially the Victoria lineage, contributes to seasonal outbreaks [6]. Surveillance data from neighboring countries, including Saudi Arabia and Egypt, reveal similar trends, with influenza A predominating and influenza B showing regional variations in prevalence [7,8,9,10].

In the broader MENA region, influenza is a leading cause of hospital admissions for respiratory infections [2,11,12]. Studies from Saudi Arabia indicate that A(H1N1) pdm09 was the most frequently detected subtype during the 2009–2011 pandemic and has continued to circulate as the dominant strain in subsequent years [7,13]. Research by Althaqafi et al. (2021) [8] found that A(H1N1) pdm09 and A(H3N2) were the most common influenza strains in Saudi Arabia, with A(H3N2) being more prevalent in the general population during winter. Similarly, in Egypt, influenza A viruses have been the most commonly detected in recent seasons, while influenza B (Victoria and Yamagata lineages) has been observed at lower rates but remains a contributor to seasonal outbreaks [9,10].

Given the ongoing challenges in controlling influenza transmission in the MENA region, sustained surveillance and improved vaccination efforts are critical to mitigating influenza-related morbidity and mortality. Understanding the transmission dynamics of these viruses in Jordan and neighboring countries provides essential insights for informing public health interventions. The aim of this study is to understand the circulation patterns of influenza viruses in Jordan, providing critical insights into the dynamics of influenza and informing public health strategies in the country.

## 2. Materials and Methods

### 2.1. Cohort Description

This study was conducted across three hospitals in urban Jordan, Prince Hamza Hospital (Amman), Irbid Specialty Hospital (Irbid), and Jabal Al-Zaytoun Hospital (Zarqa), from November 2021 to April 2023. Participants, both children and adults, presenting with upper respiratory tract infection (URTI) symptoms in emergency departments or hospital admissions were included. Common symptoms included fever, sore throat, runny nose, and exertion. Patients with malignancies or terminal ICU illnesses were excluded.

Nasopharyngeal specimens were collected using flocked swabs (Zybio, Chongqing, China) and stored in transport medium at −20 ˚C until nucleic acid extraction. Ethical approval was granted by the Institutional Review Board of The Hashemite University (Approval No. 3/7/2020/2021) and Prince Hamza Hospital (Approval No. MH/517/2022). Written informed consent was obtained from participants or their legal guardians. Patient data, including demographics, clinical presentations, and medical history, were securely collected through a structured questionnaire.

### 2.2. Nucleic Acid Extraction and Multiplex Respiratory Panel

Nasopharyngeal swabs were collected from 458 patients and transported to the laboratory in an ice box for further analysis. Total nucleic acid was extracted from these samples using the QIAamp MinElute Virus Spin Kit (Qiagen, Hilden, Germany). To identify the pathogens responsible for respiratory symptoms, a comprehensive multiplex real-time RT-PCR was performed using the FTD Resp33 Panel (Fast-track Diagnostics, Luxembourg, Germany), as previously described [14,15]. This assay detects a wide range of 33 respiratory pathogens, including multiple influenza viruses (A, B, C), coronaviruses, parainfluenza viruses, rhinoviruses, respiratory syncytial viruses, adenoviruses, enteroviruses, and key bacterial species such as *Mycoplasma pneumoniae* and *Streptococcus pneumoniae*, as well as the fungus *Pneumocystis jirovecii*. The PCR reactions were prepared according to the manufacturer’s protocol, using 10 μL of extracted nucleic acid in a 50 μL reaction volume. Thermal cycling involved reverse transcription, initial denaturation, and 40 cycles of amplification. A positive result was defined by a sigmoidal amplification curve with a cycle threshold (Ct) value below 35. Internal, positive, and negative controls provided by the manufacturer were included in every run to ensure the accuracy and reliability of the results.

### 2.3. Subtype Identification and Analysis of Influenza Viruses

To identify and analyze influenza A and B subtypes, primers specific to the hemagglutinin (HA) and neuraminidase (NA) genes were selected based on the WHO recommendations for molecular detection. These primers targeted influenza A(H1N1), A(H5N1), A(H3N2), and the Victoria and Yamagata lineages of influenza B. Detailed information on subtypes, target genes, primer names, and sequences is provided in Table S1. Conventional PCR was performed using these primers to amplify the specific gene regions of the influenza viruses. PCR products were then subjected to gel electrophoresis on a 1.5% agarose gel and visualized under UV light. Positive and negative controls were included to ensure result accuracy. The presence of a band matching the expected size confirmed the presence of the corresponding influenza virus subtype.

### 2.4. Statistical Analysis

Statistical analysis was performed using IBM SPSS Statistics software, version 24, with the Exact Test package (IBM, New York, USA). Descriptive statistics were used to summarize patient characteristics, focusing on frequency distributions and proportions. The Pearson Chi-Square test was applied to assess differences between study groups, with statistical significance defined as *p* ≤ 0.05.

## 3. Results

### 3.1. Demographic of Study Participants

The study included 458 participants, with 262 males (57.2%) and 196 females (42.8%). In the IAV group, 76.7% were male, while in the IBV and ICV groups, 64.5% and 52.0% were male, respectively. The gender distribution did not differ significantly between groups (*p* = 0.107). Participants ranged in age from infancy to over 70, with 19.0% aged 0–5 years, 17.5% aged 6–18 years, 26.6% aged 19–30 years, 28.2% aged 31–50 years, and 8.7% above 50 years. In the IAV group, 65.1% were aged 19–30 years, while the IBV group had the largest proportions in the 6–18 years and 31–50 years age groups (30.6% each). The ICV group was predominantly 0–5 years (72.0%). Age distribution differences were statistically significant (*p* < 0.001) (Table 1).

Geographically, most participants were from Amman (60.7%), followed by Zarqa (22.1%), Irbid (13.8%), and other cities (1.5%). The IAV group had the highest proportion from Zarqa (72.1%), while most IBV participants were from Amman (59.6%). A notable 92.0% of ICV participants were from Amman, with no cases from Zarqa. Residential location differences across groups were statistically significant (*p* < 0.001). These findings highlight the sample’s geographical diversity, though most participants were concentrated in major cities.

### 3.2. Clinical Characteristics of Study Participants

During sample collection, a wide range of symptoms was reported (Table 2), varying significantly across groups (*p* < 0.001). The most common symptoms were cough (79.0%), nasal discharge/congestion (63.5%), sore throat (63.3%), headache (59.4%), myalgia (55.0%), and fever (53.9%). Cough was most frequent in IAV (88.4%), IBV (83.9%), and ICV (100%) cases. ICV cases also had higher rates of difficulty breathing (76.0%) and diarrhea (20.0%).

Most participants were healthy (80.1%), while 5.7% had hypertension, 4.6% had allergies, 4.1% had asthma, and 3.3% had diabetes. Chronic conditions were slightly more prevalent in the IAV and IBV groups than in ICV. The predominance of healthy individuals, alongside those with chronic conditions, provides a representative health profile of the study sample.

### 3.3. Distribution of Infections and Coinfections Among Study Participants

**Figure 1:**
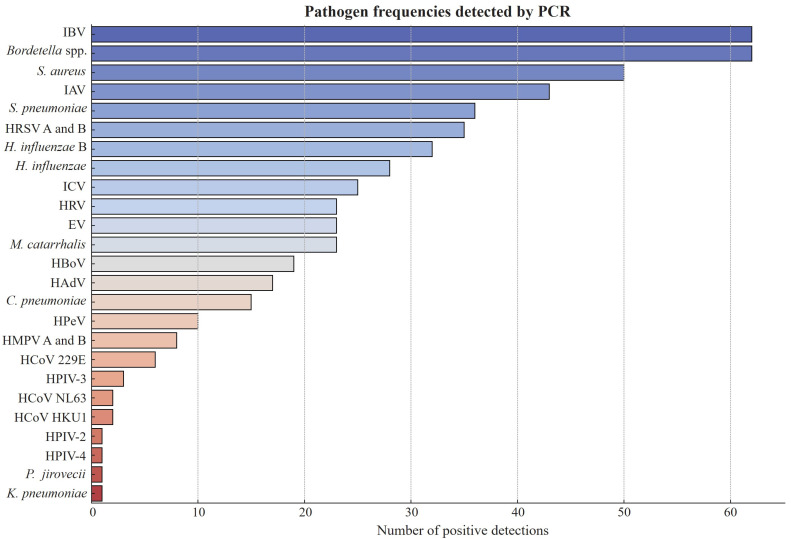
Frequencies of respiratory pathogens detected using Fast Track Diagnostics (FTD) real-time PCR kit (*n* = 458). IBV: influenza B virus; IAV: influenza A virus; HRSV: human respiratory syncytial virus; ICV: influenza C virus; HRV: human rhinovirus; EV: enterovirus; HBoV: human bocavirus; HAdV: human adenovirus; HPeV: human parechovirus; HMPV: human metapneumovirus; HCoV: human coronavirus; HPIV: human parainfluenza virus.

The study analyzed 458 participants for respiratory pathogens, revealing a broad spectrum of infections and coinfections. The most common pathogens were influenza B virus (IBV) and *Bordetella* spp., each detected in 62 participants (13.5%). *Staphylococcus aureus* infected 50 participants (10.9%), followed by influenza A virus (IAV) in 43 (9.4%) and *Streptococcus pneumoniae* in 36 (7.9%) (Figure 1). Other frequently detected pathogens included human respiratory syncytial virus (HRSV A and B) in 35 participants (7.6%), *Haemophilus influenzae* type B (32, 7.0%), and *Haemophilus influenzae* (28, 6.1%). Influenza C virus (ICV) was found in 25 participants (5.5%). Viral infections such as human rhinovirus (HRV), enterovirus (EV), and *Moraxella catarrhalis* affected 23 participants (5.0%) each.

Less common pathogens included human bocavirus (HBoV) in 19 (4.1%) and human adenovirus (HAdV) in 17 (3.7%), suggesting a lower prevalence but still having a significant role in respiratory disease. Rarely detected pathogens included *Chlamydia pneumoniae* (15, 3.3%), human parechovirus (HPeV) (10, 2.2%), and human metapneumovirus (HMPV A and B) (8, 1.7%). Coronaviruses (HCoV 229E, HCoV NL63, HCoV HKU1) were found in 2% of participants while parainfluenza viruses (HPIV-2, HPIV-4), *Klebsiella pneumoniae*, and *Pneumocystis jirovecii* were extremely rare, each detected in only one case (0.2%) (Table 2). These findings highlight the diverse etiologies of respiratory infections in the study population.

### 3.4. Coinfections with Influenza Viruses

**Figure 2:**
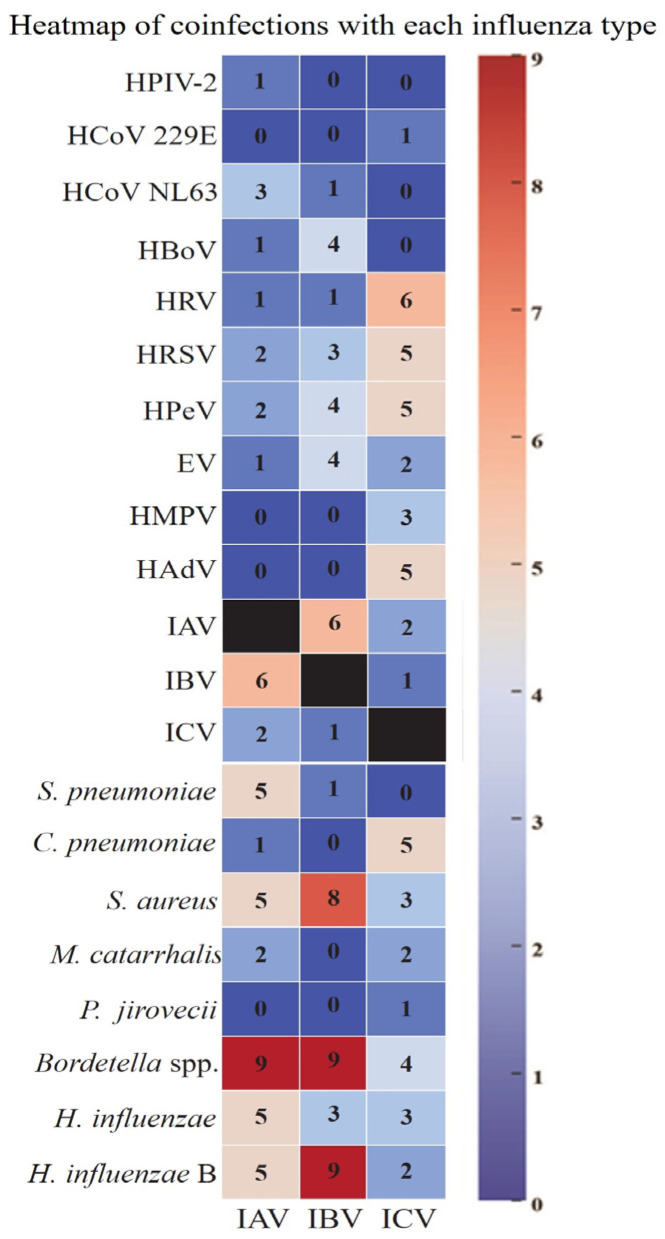
A heatmap of coinfections associated with each influenza type. This heatmap illustrates the frequency of viral and bacterial coinfections observed in patients with different types of influenza: influenza A virus (IAV), influenza B virus (IBV), and influenza C virus (ICV). The intensity of the color corresponds to the number of cases, with darker blue indicating fewer cases and red indicating a higher frequency of coinfections. The *y*-axis lists the identified pathogens, including respiratory viruses (e.g., *HPIV-2*, *HCoV 229E*, *RSV*, *HMPV*) and bacterial species (e.g., *S. pneumoniae*, *S. aureus*, *Bordetella* spp.). The numbers within the heatmap cells represent the count of cases in which a given pathogen was co-detected with the respective influenza type. The black cells represent cases of the respective influenza virus without coinfection.

The occurrence of coinfections highlighted the complex interaction between viral and bacterial pathogens in respiratory illnesses. Among the 43 IAV-positive participants, bacterial coinfections were common, with *Bordetella* spp. in nine cases, and *Staphylococcus aureus*, *Streptococcus pneumoniae*, *Haemophilus influenzae*, and *Haemophilus influenzae* type B in five cases each. Viral coinfections included IBV in six cases, HCoV 229E in three, and ICV, HPeV, and HRSV in two cases each. Single coinfections included HPIV-2, HBoV, and EV (Figure 2). IBV showed the highest rate of coinfections, with *Bordetella* spp. and *Haemophilus influenzae* type B in nine cases, *Staphylococcus aureus* in eight, and *Haemophilus influenzae* in three. Viral coinfections included IAV in six cases, with HBoV, HPeV, and EV in four cases each. These findings emphasize the importance of comprehensive testing to identify coexisting pathogens in IBV cases.

Among the 25 ICV-infected participants, viral coinfections were common, with HRV most frequent (6 cases), followed by HRSV, HPeV, and HAdV (5 cases each). Less frequent viral coinfections included HMPV (three cases), IAV and EV (two cases each), and single cases of IBV and HCoV NL63. Bacterial coinfections included *Chlamydia pneumoniae* (five cases), *Bordetella* spp. (four cases), *Staphylococcus aureus* and *Haemophilus influenzae* (three cases each), with two cases of *Haemophilus influenzae* type B and *Moraxella catarrhalis*, and a single case of *Pneumocystis jirovecii* (Figure 2). Although less common than IAV or IBV, ICV was frequently associated with multiple pathogens, highlighting the complexity of pathogen interactions in respiratory infections.

### 3.5. Subtype Distribution of Influenza A and B Viruses

**Figure 3:**
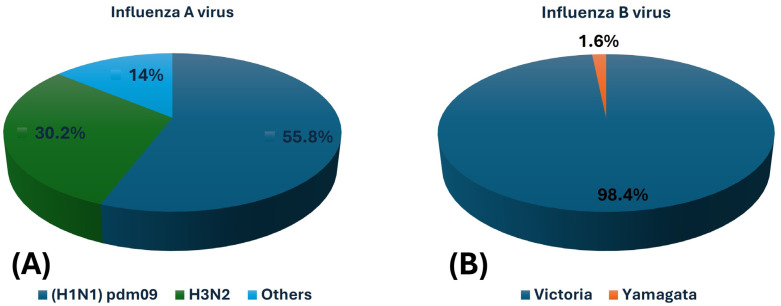
Distribution of influenza A and B virus strains detected in the study cohort. (**A**) Influenza A virus (IAV) subtypes: H1N1 (pdm09) accounted for 55.8%, H3N2 for 30.2%, and other subtypes for 14.0%. (**B**) Influenza B virus (IBV) lineages: Victoria lineage constituted 98.4% of IBV cases, while Yamagata lineage was detected in 1.6%.

This study detected 130 cases of influenza viruses, including 43 cases of influenza A (IAV), 62 cases of influenza B (IBV), and 25 cases of influenza C (ICV). For IAV, (H1N1) pdm09 was the most common subtype, found in 24 cases (55.8%), followed by H3N2 in 13 cases (30.2%). Six cases (14.0%) were attributed to other IAV subtypes not targeted by the primers. Despite testing for H5N1, none of the samples were positive for this subtype, with (H1N1) pdm09 being the dominant circulating strain during the study period (Figure 3A).

Among the 62 IBV cases, 61 (98.4%) were of the Victoria lineage, while only 1 (1.6%) was of the Yamagata lineage (Figure 3B). The dominance of the Victoria lineage reflects a strong seasonal prevalence, aligning with global trends of declining Yamagata circulation. This finding is important for guiding vaccine formulation and public health strategies.

## 4. Discussion

This study investigated the epidemiology and genetic variability of influenza viruses circulating in Jordan during the 2021–2023 seasons, alongside a high prevalence of Bordetella spp. These updated insights, nearly a decade after previous research, highlight the need for continuous surveillance to guide public health strategies and vaccine development. Moreover, the post-pandemic easing of non-pharmaceutical interventions (NPIs) influenced influenza activity globally. While the 2021–2022 season saw an increase in cases, levels remained below pre-pandemic numbers [16,17]. In some regions, abrupt surges in influenza activity were reported as restrictions were lifted, mirroring trends observed in the USA, Australia, and Brazil [18,19,20]. These variations emphasize the complex interplay among mitigation measures, behavioral changes, and viral circulation, reinforcing the need for robust surveillance systems.

Our findings indicate a resurgence of influenza cases post-COVID-19, with influenza A (9.4%), influenza B (13.5%), and influenza C (5.5%) detected in 28.4% of acute respiratory tract infection (ARTI) cases. Moreover, H1N1(pdm09) and the Victoria lineage of influenza B were predominant. Earlier studies in Jordan reported influenza as being responsible for 4.1% of ARTI cases in children under two years old (2010–2013) [2]. Regionally, influenza A(H1N1) pdm09 was the predominant strain in 2016/2017, while influenza B surged in specific years [2,21]. Influenza A and B were more prevalent in older children and young adults, whereas influenza C primarily affected children under five years old. The highest seroprevalence was observed in individuals aged 19–30 years for H3N2 and 6–18 years for H1N1(pdm09), aligning with regional and global patterns [10,22]. The shifting subtype predominance, as observed in Sri Lanka, underscores the evolving nature of influenza transmission, necessitating adaptive vaccination strategies [23].

In Lebanon, influenza A predominated in two of three seasons, with A/H3N2 dominant in 2016–2017, co-circulating with A/H1N1 in 2017–2018, and A/H1N1 prevailing in 2018–2019. Influenza B initially included both Yamagata and Victoria lineages, but Yamagata declined over time [24]. Similarly, studies from Saudi Arabia and Egypt frequently report H1N1 as the dominant influenza A subtype, which is consistent with the 55.8% prevalence in our study [10,25]. The predominance of the Victoria lineage of influenza B aligns with global trends, as Yamagata has become increasingly rare post-pandemic [26,27,28].

This study also identified a notable Bordetella spp. prevalence (13.5%) among influenza-positive cases, a finding rarely reported regionally. This underscores the importance of comprehensive diagnostic testing, given the role of bacterial coinfections in influenza-related morbidity and mortality. In Western countries, bacterial coinfections primarily involving *Staphylococcus aureus*, *Streptococcus pneumoniae*, and *Haemophilus influenzae* are well documented contributors to severe influenza outcomes [29,30]. Our results highlight the need for integrated viral and bacterial diagnostic approaches to improve patient management.

We also found that viral coinfections were also frequent, with influenza B cases showing the highest rates of co-detection. Common viral co-pathogens included human parechovirus, enterovirus, and human bocavirus. These findings align with reports from Turkey, Iran, and Latvia, demonstrating the complex interactions between respiratory viruses [31,32,33]. The persistence of certain viral strains and the potential for prolonged shedding highlight the importance of molecular diagnostic precision in differentiating active infections from incidental detections.

The predominance of H1N1(pdm09) and the Victoria lineage of influenza B in our study underscores the importance of aligning local vaccine formulations with the WHO recommendations [34]. The increasing rarity of the Yamagata lineage post-pandemic further supports the need for targeted vaccination strategies. Low influenza vaccine coverage remains a significant concern in Jordan, with recent studies indicating suboptimal uptake among both the general population and healthcare providers [4,35]. Strengthening public health messaging and increasing vaccine accessibility, particularly among school-aged children, could mitigate community transmission and reduce disease burden.

## 5. Study Limitations

Our study has several limitations. The absence of officially published influenza surveillance data from the Jordan Ministry of Health during the study period restricts direct comparisons with national trends. While the study provides valuable insights into influenza subtypes, the lack of whole-genome sequencing for all isolates limits a comprehensive analysis of viral evolution. Also, the lack of vaccination history and immune status hindered the assessment of vaccine effectiveness and immune susceptibility. Additionally, the study coincided with the easing of COVID-19 restrictions, making it difficult to distinguish pandemic-related effects from natural seasonal variations in influenza transmission.

## 6. Conclusions

This study identified IAV H1N1 (pdm09) and IBV Victoria lineage as the predominant strains in Jordan’s 2021–2023 influenza seasons, mainly affecting adults aged 19–50 in urban areas. Frequent bacterial coinfections, particularly with *Staphylococcus aureus* and *Haemophilus influenzae* type B were observed. Strengthening vaccination efforts for high-risk adults, expanding multiplex pathogen testing beyond Amman, and reinforcing antibiotic stewardship are crucial. Additionally, a nationwide influenza reporting system would improve surveillance and guide public health policies.

## Figures and Tables

**Table 1:** Demographic characteristics of study participants across IAV, IBV, and ICV groups.

Variable	N = 458 (%)	IAV N = 43 (%)	IBV N = 62 (%)	ICV N = 25 (%)	*p* Value
Gender Male Female	262 (57.2) 196 (42.8)	33 (76.7) 10 (23.3)	40 (64.5) 22 (35.5)	13 (52.0) 12 (48.0)	0.107
Age 0–5 6–18 19–30 31–50 >50	87 (19.0) 80 (17.5) 122 (26.6) 129 (28.2) 40 (8.7)	4 (9.3) 2 (4.7) 28 (65.1) 8 (18.6) 1 (2.3)	2 (3.2) 19 (30.6) 15 (24.2) 19 (30.6) 7 (11.4)	18 (72.0) 4 (16.0) 2 (8.0) 1 (4.0) 0	<0.001
Residential city Amman Zarqa Irbid Others	278 (60.7) 101 (22.1) 63 (13.8) 7 (1.5)	7 (16.3) 31 (72.1) 2 (4.7) 3 (6.9)	37 (59.6) 17 (27.4) 7 (11.4) 1 (1.6)	23 (92.0) 0 1 (4.0) 1 (4.0)	<0.001
Educational level Preschool Primary school Bachelor’s Master’s PhD	91 (19.9) 177 (38.6) 178 (38.9) 7 (1.5) 5 (1.1)	6 (14.0) 20 (46.5) 17 (39.5) 0 0	2 (3.2) 33 (53.1) 24 (38.7) 0 3	19 (76.0) 4 (16.0) 2 (8.0) 0 0	<0.001 NA

**Table 2:** Clinical characteristics of study participants across IAV, IBV, and ICV groups.

Variable	N = 458 (%)	IAV N = 43 (%)	IBV N = 62 (%)	ICV N = 25 (%)	*p* Value
**Symptoms** **Cough** **Nasal discharge** **Sore throat** **Headache** **Myalgia** **Fever** **Chills** **Difficulty breathing** **Nausea** **Vomiting** **Diarrhea**	362 (79.0) 291 (63.5) 290 (63.3) 272 (59.4) 252 (55.0) 247 (53.9) 217 (47.4) 214 (46.7) 115 (25.1) 115 (25.1) 48 (10.5)	38 (88.4) 25 (58.1) 33 (76.7) 28 (65.1) 28 (65.1) 24 (55.8) 24 (55.8) 25 (58.1) 6 (14.0) 6 (14.0) 1 (2.3)	52 (83.9) 45 (72.6) 47 (75.8) 43 (69.4) 32 (51.6) 31 (50.0) 32 (51.6) 25 (40.3) 12 (19.4) 12 (19.4) 7 (11.3)	25 (100) 18 (72.0) 13 (52.0) 4 (16.0) 4 (16.0) 12 (48.0) 4 (16.0) 19 (76.0) 12 (48.0) 12 (48.0) 5 (20.0)	<0.001
**Health status** **Healthy** **Hypertension** **Allergy** **Asthma** **Diabetes** **Heart disease** **Other illnesses**	367 (80.1) 26 (5.7) 21 (4.6) 19 (4.1) 15 (3.3) 5 (1.1) 5 (1.1)	35 (81.4) 1 (2.3) 3 (6.9) 2 (4.7) 0 0 2 (4.7)	49 (79.0) 4 (6.5) 3 (4.8) 1 (1.6) 1 (1.6) 0 4 (6.5)	24 (96.0) 0 0 1 (4.0) 0 0 0	NA

## Data Availability

The original contributions presented in this study are included in the article/Supplementary Materials. Further inquiries can be directed to the corresponding author(s).

## Supplementary Materials

The following supporting information can be downloaded at: https://www.mdpi.com/article/10.3390/pathogens14040365/s1, Table S1: List of primers used for the subtyping of Influenza A and B viruses.
