# Supplementary material for: Patterns of the Circulation of Influenza in a Targeted Jordanian Subpopulation from November 2021 to April 2023
Source: Pathogens. 2025 Apr 8;14(4):365. doi: 10.3390/pathogens14040365 (PMC12030556; doi:10.3390/pathogens14040365)
Supplement: Supplementary file 1 [file pathogens-14-00365-s001.zip › pathogens-3488987-supplementary.pdf]

Table S1. List of primers used for the subtyping of Influenza A and B viruses. The table includes the primer names, target genes, and the 5'–3' sequences.

Type/Subtype	Gene Fragment	Primer	Sequence
Influenza A(H1N1)pdm09	HA	HKU-SWF	TGAGCTCAGTGTCATCATTTGA
		HKU-SWR	TGCTGAGCTTTGGGTATGAA
Influenza A(H5N1)	HA	H5-918F	CCARTRGGKGCKATAAAYTC
		H5-1166R	GTCTGCAGCRTAYCCACTYC
Influenza A(H5N1)	HA	H5-248-270F	GTGACGAATTCATCAATGTRCCG
		H5-671-647R	CTCTGGTTTAGTGTTGATGTYCCAA
Influenza A(H3N2)	HA-5’ (H3)	H3A1F6	AAGCAGGGGATAATTCTATTAACC
		H3A1R1	GTCTATCATTCCCTCCCAACCATT
	HA-3’ (H3)	H3A1F3	TGCATCACTCCAAATGGAAGCATT
		HARUc	ATATCGTCTCGTATTAGTAGAAACAAGGGTGTTTT
	NA-5’ (N2)	NAFUc	TATTGGTCTCAGGGAGCAAAAGCAGGAGT
		H3N2R1095	TCATTTCCATCATCRAAGGCCCA
	NA-3’ (N2)	N2F387	CATGCGATCCTGACAAGTGTTATC
		NARUc	ATATGGTCTCGTATTAGTAGAAACAAGGAGTTTTTT
Former seasonal Influenza A(H1N1)	HA-5’(H1)	THAF2	GCAGGGGAAAATAAAAACAACC
		SPHAR11	TATTTTGGGCACTCTCCTATTG
	HA-3’(H1)	H1HAF552	TACCCAAACCTGAGCAAGTCCTAT
		HARUc	ATATCGTCTCGTATTAGTAGAAACAAGGGTGTTTT
	NA-5’(N1)	H1N1F6	AGCAGGAGATTAAAATGAATCCAA
		NASPR10	CCTTCCTATCCAAACACCATT
	NA-3’(N1)	N1F741	ATAATGACCGATGGCCCGAGTAAT
		NARUc	ATATGGTCTCGTATTAGTAGAAACAAGGAGTTTTTT
Influenza B Victoria lineage	HA	Bvf224	ACATACCCTCGGCAAGAGTTTC
		Bvr507	TGCTGTTTTGTTGTTGTCGTTTT
Influenza B Yamagata lineage	HA	BYf226	ACACCTTCTGCGAAAGCTTCA
		BYr613	CATAGAGGTTCTTCATTTGGGTT
